# 

**Published:** 1844-07

**Authors:** 


### BRITISH AND FOREIGN

### MEDICAL REVIEW

OR

### QUARTERLY JOURNAL

OF

### PRACTICAL MEDICINE AND SURGERY

STATE OF LOUISIANA CHARITY HOSPITAL * MEDICAL LIBRARY
JUSTICE
LIBRARY OF THE
Orleans Parish Medical Society

EDITED BY

JOHN FORBES M.D. F.R.S. F.G.S.

---

VOL. XVIII

JULY—OCTOBER 1844

LONDON
JOHN CHURCHILL PRINCES STREET SOHO
MDCCCXLIV.

C. AND J. ADLARD, PRINTERS, BARTHOLOMEW CLOSE.

### BOOKS RECEIVED FOR REVIEW.

1. Traité complet de l'Anatomie, de Physiologie, et de la Pathologie du Système Nerveux Cérébro-spinal. Par M. Foville, M.D., &c. Prem. Part, avec un Atlas de xxiii Planches.—Paris, 1844. 8vo, pp. 676.

2. An Essay on Pneumo-Thorax. By H. M. Hughes, M.D.—London, 1844. 8vo, pp. 37.

3. The actual Process of Nutrition in the Living Structure, demonstrated by the Microscope, &c. 5s.

4. Prize Essay on the Nature and Objects of Medical Science. By P. H. Williams, M.D.—London, 1844. 8vo, pp. 52.

5. Medicines, their Uses and Mode of Administration. By J. M. Neligan, M.D., Lecturer in the Dublin School of Medicine.—Dublin, 1844. 8vo, pp. 432. 12s.

6. Geology. By D. T. Ansted, F.R.S. Part I. London, 1844. 5s.

7. A History of British Fossil Mammalia and Birds. By R. Owen, F.R.S. &c. Part I.—London, 1844. 2s. 6d.

8. Outlines of Pathology and Practice of Medicine. By W. P. Alison, M.D., &c. Part II.—Edinb. 1844. 6s.

9. On Conical Cornea. By J. H. Pickford, M.D., —Dublin, 1844. 8vo, pp. 16.

10. Illustrations of the Theory and Practice of Ventilation, &c. By D. B. Reid. M.D., F.R.S.—London, 1844. 8vo, pp. 541. 15s.

11. The Results of the great Operations of Surgery, during a Practice of 22 Years; showing a high rate of success, at the Liverpool Infirmary. By J. P. Halton.—Liverpool, 1844. 8vo, pp. 34.

12. Reply from the Surgeons of the Liverpool Northern Hospital to a Pamphlet published by J. P. Halton—London, 1844. 8vo, pp. 39.

13. General Report of the Royal Hospitals of Bridewell and Bethlem. London, 1844. 8vo, pp. 81. 7s. 6d.

14. Chemistry, as exemplifying the Wisdom and Beneficence of God. By George Fownes, PH.D.—London, 1844. 8vo, pp. 184.

15. The principal Offices of the Brain and other Centres. By J. Swan.—Lond. 1844. 8vo, pp. 31.

16. Scrofula; its Nature, Causes, and Treatment, &c. By W. Tyler Smith, M.B.—London, 1844. 8vo, pp. 172. 7s.

17. Practical Observations on the Prevention, Causes, and Treatment of Curvatures of the Spine. By S. Hare, Surgeon. 2d edition, revised and enlarged. London, 1844. 8vo, pp. 177.

18. An Essay on the Tongue in Functional Derangements of the Stomach, Bowels, &c. By E. Williams, M.B. Cant.—Lon. 1843. 8vo, pp. 119.

19. A Treatise on the Use of the Sympathetic Nerve and its Ganglions. By T. B. Procter, M.D. Lond. 1844. 4to, pp. 47.

20. The Chemical and Physiological Balance of Organic Nature. By M. J. Dumas and M. J. B. Bossingault. 3d ed. Lond. 1844. 12mo pp. 156.

21. Mesmerism and its Opponents; with a Narrative of Cases. By G. Sandby, jun., A.M., Vicar of Hinton.—London, 1844. 8vo, pp. 278.

22. A Dictionary of Medical Science. By R. Dunglison, M.D. 4th ed.—Philadelphia, 1844. 8vo, pp. 771.

23. Homœopathy Unmasked. By A. Wood, M.D.—Edinburgh, 1844. 8vo, pp. 196. 4s. 6d.

24. A Manual of the Practice of Medicine; the result of fifty years' experience. By W. C. Hufeland. Translated by C. Bruchausen, and R. Nelson, M.D.—London, (New York ?) 1844. 8vo, pp. 630.

25. The three Cardinal Means of the Art of Healing. By W. C. Hufeland. — New York. 8vo, pp. 79.

26. On Dysmenorrhea and other Uterine Affections in connexion with Derangement of the Assimilating Functions. By Edward Rigby, M.D.—London, 1844. 8vo, pp. 138.

27. Remarks on the use of Vivisection as a means of Scientific Research. By R. Jameson. —London, 1844. 8vo, pp. 52.

28. Facts and Observations on the Sanitary State of Glasgow during the last Year. By R. Perry, M.D.—Glasgow, 1844. 8vo. pp. 37.

29. Chemistry simplified in its application to the Testing of Alkalies, &c. By Andrew Ure, M.D. F.R.S. 8vo, pp. 84.

30. The Nature and Treatment of Deafness and Diseases of the Ear. By W. Dufton, M.R.C.S. —London, 1844. 8vo, pp. 118. 4s.

31. On the Nature and Treatment of some of the more important Diseases, Medical and Surgical. By J. C. Hall, M.D. 2d edition, enlarged. —London, 1844. 8vo, pp. 247. 7s. 6d.

32. First Lines for Chemists and Druggists, preparatory to examination, &c. By J. Steggall, M.D.—London, 1844. 18mo. pp. 170. 3s. 6d.

33. Remarks on the Efficacy of Matico as a Styptic and Astringent. By Thos. Jeffreys, M. D. 2d edition.—Liverpool, 1844. 8vo, pp. 37.

